# Workshop on Implementation Science and Digital Therapeutics for Behavioral Health

**DOI:** 10.2196/17662

**Published:** 2021-01-28

**Authors:** Sarah E Lord, Aimee N C Campbell, Mary F Brunette, Leonardo Cubillos, Sophia M Bartels, William C Torrey, Ardis L Olson, Steven H Chapman, John A Batsis, Daniel Polsky, Edward V Nunes, Katherine M Seavey, Lisa A Marsch

**Affiliations:** 1 Center for Technology and Behavioral Health Geisel School of Medicine at Dartmouth College Lebanon, NH United States; 2 Department of Psychiatry Dartmouth Hitchcock Medical Center Lebanon, NH United States; 3 Department of Psychiatry Columbia University Irving Medical Center New York, NY United States; 4 Geisel School of Medicine at Dartmouth College Lebanon, NH United States; 5 Department of Pediatrics Dartmouth Hitchcock Medical Center Lebanon, NH United States; 6 Department of Medicine Dartmouth Hitchcock Medical Center Lebanon, NH United States; 7 The Dartmouth Institute for Health Policy & Clinical Practice Dartmouth College Lebanon, NH United States; 8 Department of Health Policy and Management Bloomberg School of Public Health Johns Hopkins University Baltimore, MD United States

**Keywords:** mHealth, mobile health, digital health, telemedicine, eHealth, behavioral sciences, substance-related disorders, mental health, implementation science

## Abstract

Digital therapeutics can overcome many of the barriers to translation of evidence-based treatment for substance use, mental health, and other behavioral health conditions. Delivered via nearly ubiquitous platforms such as the web, smartphone applications, text messaging, and videoconferencing, digital therapeutics can transcend the time and geographic boundaries of traditional clinical settings so that individuals can access care when and where they need it. There is strong empirical support for digital therapeutic approaches for behavioral health, yet implementation science with regard to scaling use of digital therapeutics for behavioral health is still in its early stages. In this paper, we summarize the proceedings of a day-long workshop, “Implementation Science and Digital Therapeutics,” sponsored and hosted by the Center for Technology and Behavioral Health at Dartmouth College. The Center for Technology and Behavioral Health is an interdisciplinary P30 Center of Excellence funded by the National Institute on Drug Abuse, with the mission of promoting state-of-the-technology and state-of-the-science for the development, evaluation, and sustainable implementation of digital therapeutic approaches for substance use and related conditions. Workshop presentations were grounded in current models of implementation science. Directions and opportunities for collaborative implementation science research to promote broad adoption of digital therapeutics for behavioral health are offered.

## Introduction

The research-to-practice gap is a significant challenge to the health care field, and translation of evidence-based practices for substance use and mental health conditions is no exception [1-3]. Recent data indicate only 11% of people in the United States with a substance use condition received treatment, and fewer than half (43%) of adults with a mental health condition received treatment [4]. Technology offers great promise for addressing many of the barriers to adoption of evidence-based treatments for behavioral health conditions.

Digital behavioral health therapeutics, delivered by way of familiar and nearly ubiquitous platforms such as the web, smartphone mobile applications, text messaging, video conferencing, and remote monitoring, can transcend the time and geographic boundaries of traditional settings so that broad audiences can access evidence-based care when and where they need it. The flexibility of technology and incorporation of mixed media (eg, text, audio, video, animation, avatars, virtual reality) allow for tailoring of content to meet individual needs across a range of learning styles to promote engagement, a key marker of intervention success [5]. Digital therapeutic tools improve consistency of delivery of key intervention components, enhancing fidelity, and usage data allow clinicians and researchers to evaluate whether users adhere to recommended doses. The broad accessibility of digital devices can be used to empower individuals to be more actively self-directed in their care.

There is strong and growing evidence for digital therapeutic approaches for substance use and mental health conditions [6-8]. The empirical literature includes studies of digital therapeutic approaches across the care continuum for a range of patient and client populations and in diverse settings. For example, there are a number of evidence-based digital screening and assessment tools that represent translation of existing validated screening and assessment instruments to web-based or mobile application platforms [9-13]. These tools have been evaluated in studies across a range of settings, including addiction and mental health treatment and primary care settings. Emerging empirical work also highlights the promise of wearable technologies and remote sensing for passive identification of symptoms such as drug craving, stress, depression, and schizophrenia [14-18]. For example, innovations in machine learning and digital phenotyping were used to predict substance use risk [19] and opioid overdose in community regions [20]. The literature highlights the exciting opportunities of digital screening and assessment strategies to identify substance use and mental health symptoms as a first step to timely delivery of appropriate treatment.

There is also strong and growing support for the effectiveness of digital treatment approaches for substance use and mental health conditions, across a range of substances (eg, tobacco, alcohol, marijuana, cocaine, opioids) and mental health diagnoses (eg, depression, anxiety, posttraumatic stress disorder, schizophrenia, bipolar disorder) and across a range of adolescent and adult populations. Digital therapeutic tools developed with foundations in evidence-based principles such as motivational enhancement, behavioral and cognitive behavioral therapies, and mindfulness have demonstrated positive impact on symptom management and behavior change for substance use [21-33] and mental health conditions [34-43]. There is also good evidence for digital therapeutic approaches to support recovery following treatment [44-46].

Yet, despite promising impact, digital behavioral health therapeutics have yet to see widespread uptake in practice. The implementation science with regard to scaling adoption and sustaining implementation of digital therapeutics for behavioral health is still in its early stages. The Center for Technology and Behavioral Health (CTBH) is a P30 “Center of Excellence” supported by the National Institute on Drug Abuse with the mission to promote state-of-the-technology and state-of-the-science related to development, evaluation, and sustainable implementation of digital therapeutic approaches for substance use and related behavioral health conditions. Three CTBH Cores, Treatment Development & Evaluation, Emerging Technologies & Data Analytics, and Dissemination & Implementation (D&I) Science, bring together a diverse team of national and international researchers with expertise in addiction science, behavioral health treatment, computer science and engineering, health economics, health services delivery, and implementation science. To promote transdisciplinary research collaborations, CTBH hosted a series of day-long workshops focused on Core missions, with past workshops in Emerging Technologies & Data Analytics [47] and Treatment Development & Evaluation [48]. In this paper, we summarize a workshop hosted by the D&I Core focused specifically on implementation science as related to digital therapeutics for behavioral health. In this paper, we summarize the proceedings of the workshop and offer directions for collaborative research to propel implementation science and promote broad adoption of digital therapeutics for behavioral health.

## Methods

The day-long workshop, “Implementation Science and Digital Therapeutics,” was conducted in December 2018 on the campus of Dartmouth College, and 9 CTBH-affiliated scientists were invited to present their work related to digital therapeutics for behavioral health. The presenters were primarily clinical researchers, representing addiction medicine, psychiatry, pediatrics, internal medicine, and behavioral economics. Presenters were encouraged to use implementation science models (eg, Consolidated Framework for Implementation Research [CFIR] [49] and Outcomes for Implementation Research [OIR] [50]) as guiding frameworks for the presentations and discussions. All of the studies presented at the workshop were approved by an institutional review board or were exempt from review (ie, quality improvement project).

Local CTBH faculty, trainees, students, and staff from all Center Cores at Dartmouth College were invited to attend the campus-based workshop. Of approximately 70 invitations, 50 participants attended the all-day meeting, representing computer science, engineering, user experience and interface design, biomedical data sciences, public health, addiction medicine, psychiatry, primary care, internal medicine, digital intervention research, and health services implementation research. The workshop audience was diverse in terms of gender and race/ethnicity representation. Although the majority of the attendees and speakers were from Dartmouth College, the insights from the workshop are broadly applicable.

## Principles and Methods

### Overview of Implementation Science and Digital Therapeutics

D&I Core director Sarah Lord provided an overview of the CFIR [49] and OIR [50] as related to digital therapeutic research. The CFIR, a comprehensive framework of contextual determinants associated with successful implementation of innovations in health care settings, can readily be applied to digital behavioral health therapeutics. The CFIR organizes implementation determinants representing characteristics of the innovation (eg, ease of use and perceived usefulness of a mobile app for opioid use and depression), characteristics of individuals using the innovation (eg, self-efficacy for using a mobile app, clinician attitudes about using technology for treatment), characteristics of the setting within which the digital therapeutic is intended to be used (eg, readiness of a primary care practice to use a mobile app with patients with identified opioid use disorder and depression), characteristics of the external ecosystem (eg, limited broadband and wireless infrastructure in rural regions, electronic health record requirements), and characteristics of the implementation process (eg, planning, engagement, execution, evaluation and reflection). The OIR framework outlines 8 outcomes of implementation research, including acceptability, adoption, appropriateness, feasibility, fidelity, reach, cost, and sustainability, that can be applied to digital therapeutic research [50]. Lord described how digital approaches, by nature of technology itself (eg, consistent delivery of intervention components, usage data capture), can enhance achievement and ongoing measurement of implementation outcomes (eg, fidelity, reach) and hold promise for advancing implementation science more broadly.

**Figure 1:**
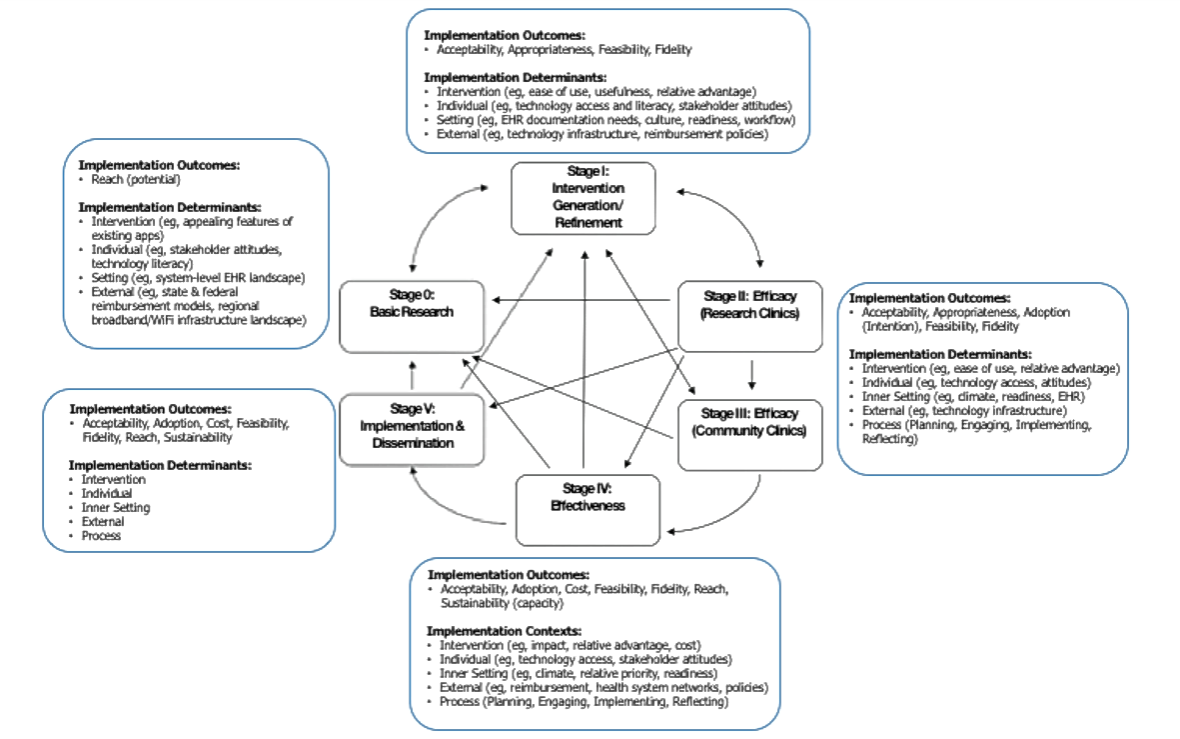
Mapping implementation-related constructs to stages of digital therapeutic development research. EHR: electronic health record.

Lord described a rationale for incorporating an implementation science perspective throughout the stages of digital therapeutic development research to maximize potency of the digital therapeutic while also optimizing implementation, building upon the National Institutes of Health Stage Model of Treatment Development [51]. Lord presented an enhancement to the model that maps salient implementation outcomes and CFIR determinants at each stage of research and outlined what could be learned about implementation at each stage and how the approach could propel translational science of digital therapeutics in systems of care (Figure 1). The enhanced model could serve as an organizing framework and common language for behavioral health scientists, data scientists, implementation scientists, and technology developers to work together on a shared mission to most expeditiously develop potent digital behavioral health therapeutic approaches that can be broadly translated into systems of care. The enhanced model suggests anchors for measurement of implementation-related constructs at each stage to promote cross-study and cross-setting common data to address questions such as, “What characteristics of digital therapeutics, individual end users, health care settings, and the larger ecosystems facilitate or impede implementation outcomes?” A compendium of existing evidence-supported implementation outcome and determinant measures that could be used in digital therapeutic research can be found on the CTBH website [52].

### Implementation in Practice: Innovations in Adaptation and Evaluation

Keynote speaker Aimee Campbell highlighted models for adapting evidence-based digital interventions and evaluating implementation in systems of care. Campbell described adaptation of the evidence-based Therapeutic Education System (TES), a digital substance use treatment program grounded in the community reinforcement approach for substance use disorder [21], for use with American Indian (AI) and Alaskan Native (AN) populations. As part of the formative work to inform adaptation, Campbell and team outlined key facilitators and barriers to implementation of TES demonstrated in a large clinical trial study in treatment settings [21]. Characteristics of the treatment setting facilitated TES implementation and intention to use TES. Clinicians’ perceived social norms regarding use of TES in the agency influenced their intentions to implement TES [53]. Clinician involvement with TES improved clinician attitudes towards digital treatment and increased patient retention and adherence [54].

In a pilot with AI and AN adults seeking treatment, participants reported that TES provided important and relevant information, but lacked cultural relevance in delivery of the information [55]. Campbell described use of the ADAPT-ITT Model [56] to guide adaptation of TES for AI and AN populations. The iterative process involved potential end users in identifying areas for adaptation to improve cultural relevance of program content and delivery. Adaptations included use of AI and AN words and slang terms, use of Native actors in video depictions, and culturally relevant representations (eg, references to and depictions of nature). The process yielded an adapted TES version that was judged by clinician end users as having higher relative advantage and compatibility with treatment provision goals relative to usual care and that was highly acceptable to representative AI and AN patient end users [57]. The work of Campbell and colleagues highlighted the important role of end user engagement in adaptation of digital therapeutics for promoting intervention relevance and adoption.

## Integrating Technology Into Practice

### Technology Tools for Tobacco Use Disorder in People With Serious Mental Health Conditions: Implementation Factors

**Figure 2:**
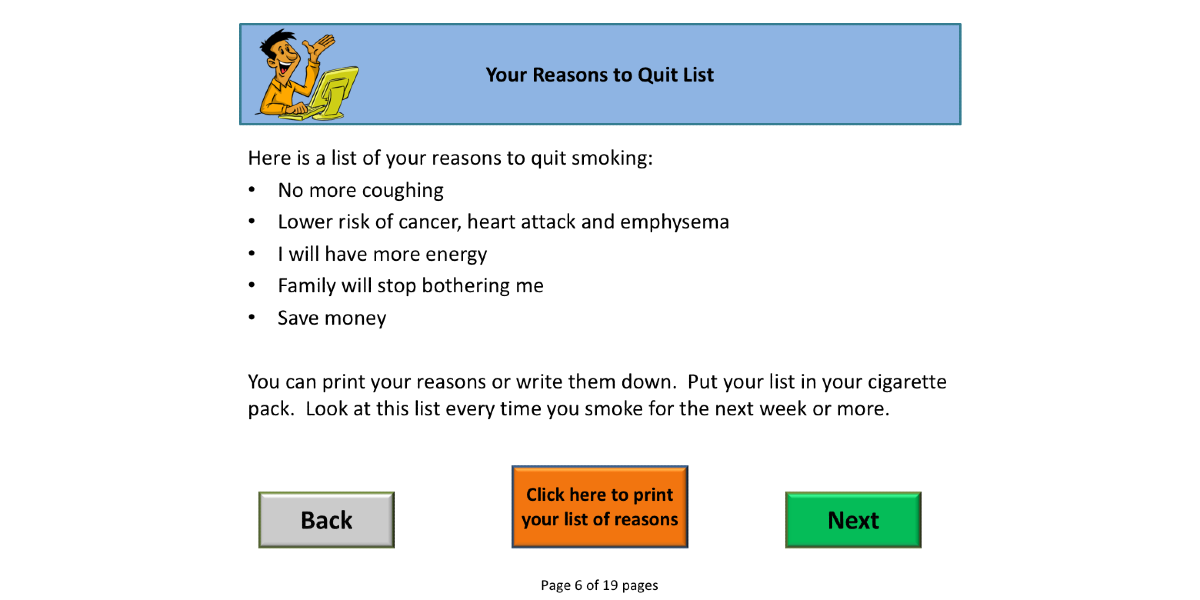
Feedback screen from the smoking cessation decision support tool, “Let’s Talk About Smoking”.

Mary Brunette described a program of research to develop and evaluate digital therapeutics for smoking among individuals with serious mental health conditions. Brunette outlined a user-centered design approach to develop a brief digital motivational intervention to promote initiation of the smoking cessation treatment, “Let’s Talk About Smoking” (Figure 2) [58,59]. The iterative approach yielded a program that was reported as easy to use by the target population, a group with cognitive impairment and generally less computer experience. The program was also highly acceptable and more appealing than standard tobacco education to the population. In a pilot study of the single-session intervention, over 45% of those who used the program proceeded to enroll in smoking cessation treatment [60].

Brunette also described results of a web-based smoking cessation program grounded in cognitive behavioral therapy, “Let’s Talk About Quitting Smoking” [33]. In pilot testing, 25% of participants reduced smoking, and 10% demonstrated bioverified abstinence at 2 months [33]. Clinician-identified barriers to ongoing implementation of the digital intervention included uncertainty about reimbursement for the intervention, lack of clinician buy-in for use of the technology, and the need for additional training and technical assistance for patient subgroups.

### Scaling Up Science-Based Mental Health Interventions in Latin America

The next set of presentations highlighted a collaborative project, funded by the National Institute for Mental Health, being conducted by investigators at CTBH and clinicians and researchers in Latin America to integrate digital therapeutic services for depression and alcohol use disorder (AUD) in primary care settings in low- and middle-income countries (LMICs) in Latin America. Leonardo Cubillos prefaced the panel with a description of a systematic review evaluating implementation of integrated mental health care in LMICs. Cubillos presented a preliminary review of 58 articles representing studies evaluating integrated mental health care in primary care settings in LMICs, with evidence to suggest that integration of mental health services improves outcomes for depression and alcohol use and is cost effective. Based on the review, Cubillos outlined a new typology of models of behavioral health integration that have been used in LMICs, including mental health care delivery by skilled and nonskilled mental health and primary care staff [61].

**Figure 3:**
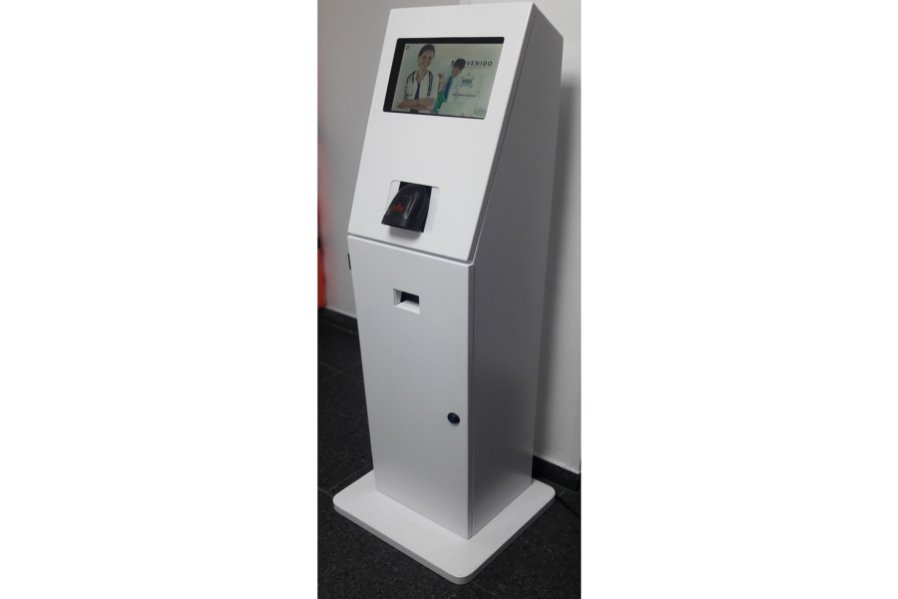
Primary care waiting room kiosk used for the screening of depression and alcohol use disorder.

**Figure 4:**
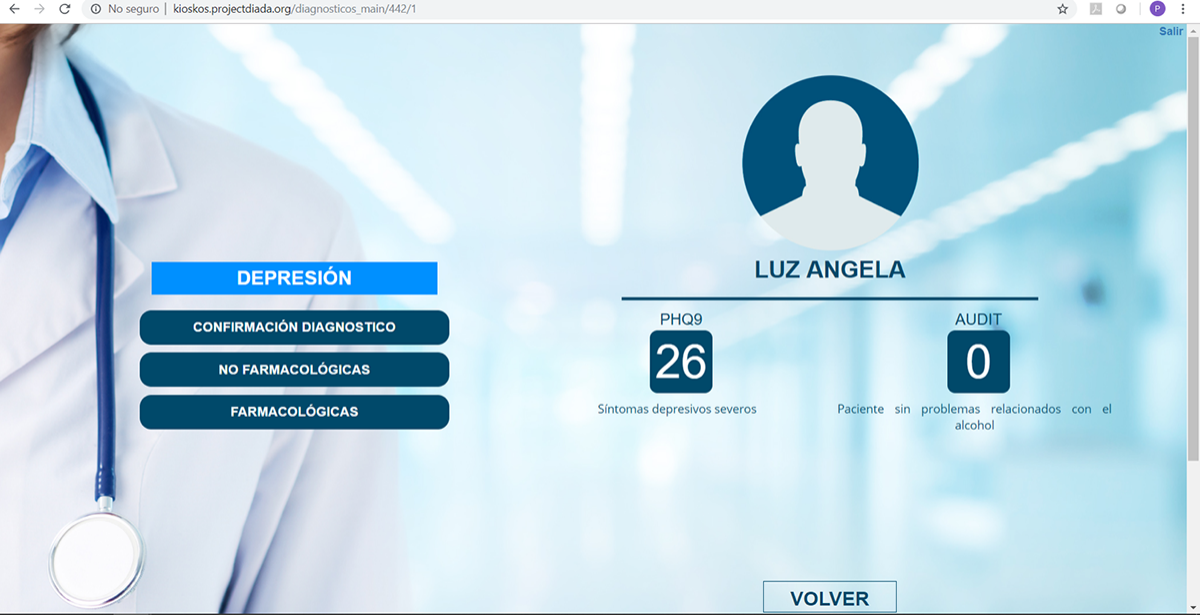
Decision support tool for primary care physicians displaying patient scores for depression and alcohol use disorder screening.

William Torrey and Sophia Bartels presented initial data from an implementation study to scale a technology-facilitated, evidence-based approach to addressing depression and AUD in primary care settings in Colombia. The researchers introduced a suite of digital tools in primary care settings that included (1) digital kiosks in the waiting rooms for patients to self-enter data to promote systematic screening for depression and AUD (Figure 3), (2) tablet-based decision support tools to guide the primary care physicians to use the screening information in their patient assessment for evaluation and diagnosis (Figure 4), and (3) a digital therapeutic for active treatment of patients found to have AUD and depression. The first phase of the implementation study was conducted in primary care settings in 6 regions of Colombia, representing urban and rural communities.

Torrey and Bartels described a methodical implementation process for the rollout of the new model of technology-driven care that included elicitation of patient, provider, and community member input through quantitative and qualitative methods to assess barriers to current processes for identifying and treating depression and AUD and to determine opportunities for integrating technology into the care workflow. Iterative engagement with primary care organization stakeholders informed strategies to build clinical capacities (eg, mental health training, technology training) and to develop technological infrastructure to support the digital components of the service suite. Implementation was evaluated with quantitative surveys and qualitative interviews to measure implementation outcomes and contextual determinants in addition to patient outcomes.

Preliminary results indicated good feasibility and acceptability of the integrated digital care model and positive perceptions of compatibility of the digital care model with current practice at the primary care settings, particularly in the urban communities in which the model was integrated. Primary care doctors increased the percentage of patients diagnosed as having depression and AUD from near 0% to 17% and 2%, respectively [62]. A flexible, supportive organizational climate and perceived fit of the intervention with the clinic practice facilitated integration. Difficulty engaging patients with AUD, low provider self-confidence for making a difference, social norms that support drinking, and physician concerns about labeling patients with a diagnosis were identified as barriers to diagnosing and treating patients in need. Poor technology infrastructure (eg, limited internet or WiFi capacity) in rural regions was also a barrier to uptake of the digital therapeutic tools. The presentations by Cubillos, Torrey, and Bartels highlighted how reverse innovations from research in low-resource countries can advance scientific knowledge for implementation of digital therapeutics in resource-limited regions of the United States.

### Integration of Digital Therapeutics in Primary Care in the United States

**Figure 5:**
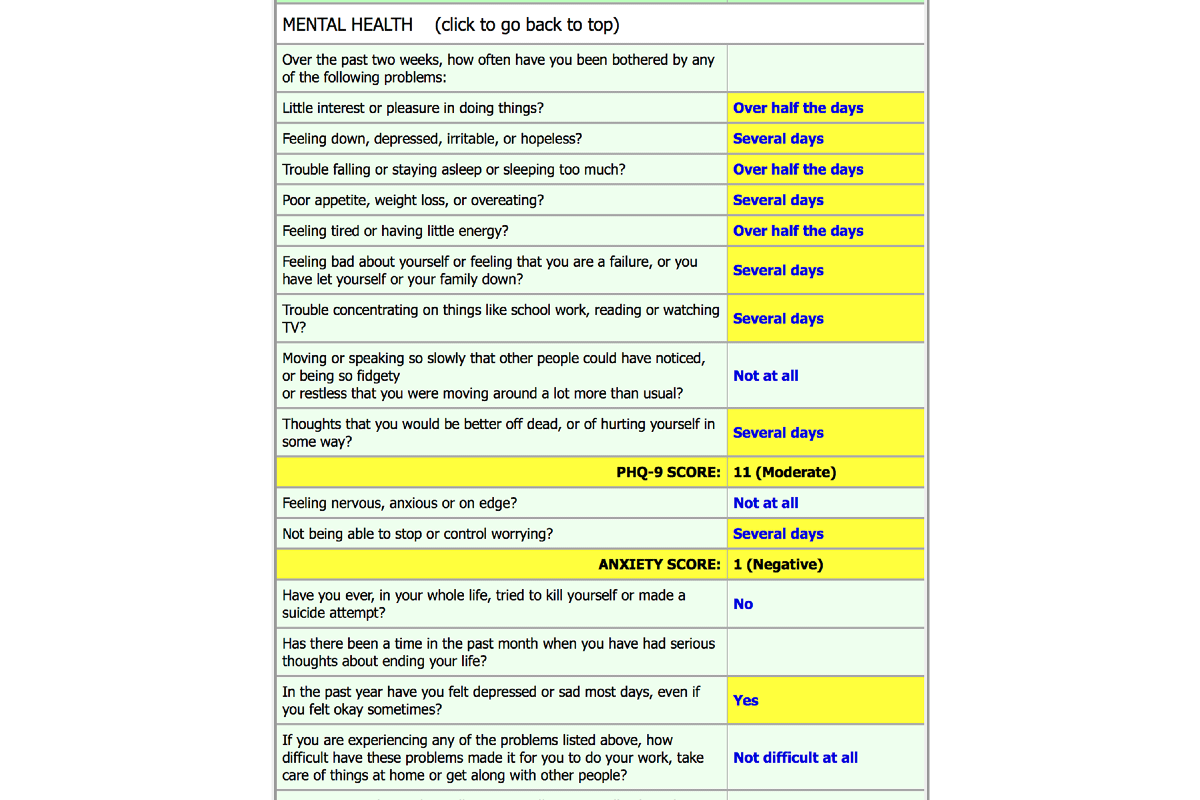
Patient-clinician collaborative review screen from DartScreen.

The next presentations highlighted efforts by clinical researchers to integrate digital therapeutics into pediatric primary care and internal medicine settings. Ardis Olson described the >13-year process involved in the development and implementation of DartScreen, a digital health risk behavior screening tool, in pediatric practices (Figure 5). DartScreen was iteratively developed by primary care clinicians and an academic pediatrician as part of the Dartmouth primary care research network [63,64]. The content and delivery approach of the screener was informed by the American Medical Association Guidelines for Adolescent Preventive Services, including (1) using validated substance use and mental health screening tools (eg, CRAFFT, PHQ-2), (2) addressing patient readiness to change for key health risks, and (3) providing easily digestible results for clinicians to access at the beginning of the patient visit (eg, summary screen of health risks with scoring and risk levels highlighted). The initial DartScreen was administered on personal digital assistants. DartScreen is now available as a standalone web-based version implemented on tablets, as well as integrated in EPIC.

The web-based version of DartScreen is being used in 13 pediatric practices throughout Vermont and New Hampshire and has reached over 20,000 adolescents, with demonstrated positive impact on adolescents’ and pediatric clinicians’ experiences of receiving and providing care. Adolescents who used DartScreen were more likely than controls to report being satisfied with their visit and being listened to by their provider and less likely to report that there was something they wanted to talk about with their provider but did not [65]. In a study of audio-recorded teen health visits before and after using DartScreen, use of DartScreen was associated with decreases in provider data gathering and counseling, increases in provider responsiveness and engagement with their patients, greater expressed attentiveness to patients, and more discussion about mental health topics [66]. Use of DartScreen was also associated with increases in teens’ reporting of psychosocial information and engagement in dialogue [66].

In a survey study of clinicians who used DartScreen in their pediatric practice workflow, clinicians rated DartScreen as highly useful in the clinical process and easy to use [67]. Facilitators to adoption of DartScreen in practices included identifying a practice champion; demonstrating DartScreen to practice administrators, clinicians, and staff; and trialing of the digital screening tool by a subset of clinicians before progressing to full adoption into office procedures. Barriers to implementation included desire for integration of the screener with electronic health records, unreliability of technology infrastructure (eg, practice internet connectivity issues), and the screening taking too long for some adolescents.

Steven Chapman described integration of the DartScreen into the Dartmouth-Hitchcock Medical Center electronic medical record (EPIC) as a component of Screening, Brief Intervention, and Referral to Treatment (SBIRT) for adolescents and young adults in primary pediatric care. A multidisciplinary team collaborated in greenbelt sessions using a Six Sigma quality improvement methodology and a Define, Measure, Analyze, Improve, Control process to implement SBIRT. All teenagers from all pediatric practices in the Dartmouth-Hitchcock system were screened using the DartScreen tool. Screening rates were regularly above 80% after implementation. Fishbone analysis examining gaps in screener completion and brief intervention delivery indicated that researchers needed to refine workflow for administration of the screener, incorporate tools and prompts for clinicians, and provide training for clinicians in motivational interviewing and brief interventions. The presentation highlighted the benefits of a systematic approach to integration of an evidence-based digital approach into practice, using an institutional quality improvement framework, leveraging electronic medical records, and using co-design methodologies to promote relevance for stakeholders and buy-in.

### Implementation Strategies for Addressing Rural Obesity Using Telehealth: Barriers, Facilitators, and Lessons Learned

Internist John Batsis described an early-stage study to evaluate a telemedicine intervention to increase reach of an evidence-based lifestyle program for adult patients with obesity. Batsis described strategies used to iteratively adapt in-person, group-based lifestyle intervention classes to be delivered remotely to individuals (not as a group) via videoconferencing, based on clinician stakeholder feedback. Evaluation of implementation of the telemedicine approach demonstrated that a large majority of participants (>93%) found the telehealth platform easy to use and useful and would use the intervention in the future. The intervention reached 8.4% of potential participants referred to the intervention. There was preliminary evidence for efficacy of the 16-week telemedicine intervention for improving body mass index and waist circumference [68].

Clinicians perceived a number of advantages of the telemedicine intervention delivery, including expanded access to individuals from rural regions for whom in-person participation was a barrier and high confidence using the telehealth platform. Implementation barriers included clinicians’ perceptions of reduction of patient-clinician rapport due to less face-to-face time, clinician fatigue from individualized delivery format to successive patients, practice-level scheduling and space constraints for delivery of the telehealth intervention, and unreliable patient technology infrastructure (eg, connectivity interruptions). Batsis outlined directions for future work to address barriers, including space and time to accommodate visits, inclusion of a one-on-one, in-person visit, and initial assessment of readiness to change to guide individualized treatment planning.

### Economic and Policy Perspectives on Implementation Science for Behavioral Health

The final presenter, health economist Daniel Polsky, discussed the value that economists bring to implementation science, including informing decisions about whether evidence-based practice should be disseminated broadly and helping to determine how incentives can induce behavior change to enable broad dissemination of evidence-based digital therapeutics and improve return on investment for funding agencies. Economists can also lend insight into when to de-implement a practice that does not work in a given setting or with a given population. Polsky highlighted how stakeholder groups and disciplines view problems from different perspectives and noted that economists can help bridge divides between researchers and stakeholder groups, such as policy makers. Stakeholders are all interested in sustainability and access to innovative treatments; there is a significant need for research to be translated in ways that are relevant to all stakeholders. Polsky encouraged digital health researchers to include economists and policy stakeholders on study teams throughout the stages of digital intervention research to optimize potential for sustainability and broad system translation of digital therapeutics.

## Discussion

To close the workshop, Edward Nunes, an addiction psychiatrist and Deputy Director for Intervention Studies in the CTBH Treatment Development and Evaluation Core, led a discussion during which panelists and workshop participants highlighted key themes and directions for future research, including accelerating evaluation through innovative development and implementation science methodologies, promoting academic-industry partnerships to accelerate evaluation and translation of digital therapeutic approaches for behavioral health, identifying strategies to promote awareness about and use of science-supported digital therapeutics, harnessing big data and predictive modeling strategies to promote implementation science for digital therapeutics, and addressing system context barriers.

### Accelerate Evaluation Through Innovative Development and Implementation Science Methodologies

Workshop presentations highlighted the importance of user-centered development processes and engagement of the range of potential end users and stakeholders (eg, patients, clinicians, economists, policy makers) across all stages of digital intervention research to optimize potential for adoption and successful implementation. For example, the presentations by Olson and Chapman describing integration of the DartScreen in pediatric care demonstrated how early involvement of health system stakeholders, such as through practice-based research networks and Six Sigma quality improvement processes, promoted high buy-in from clinicians and alignment of the digital approach with clinical workflow to facilitate implementation sustainability of the screener in pediatric settings.

The use of small pilot studies to optimize intervention design and implementation with end users encourages engagement and can help avoid large investments in development and research only for an intervention to not be practically implementable. The broad accessibility of digital devices offers opportunities for trialing of digital therapeutics with patients and clients in diverse care settings to identify tailored implementation support strategies. The ability to easily trial an intervention promotes successful adoption and implementation [49].

The flexibility of technologies also allows for use of innovative study designs, such as factorial and microrandomized trials, to test different intervention components and the impact of different implementation strategies for promoting use of a given digital therapeutic [69-71]. Hybrid study designs that support evaluation of clinical effectiveness as well as implementation can more efficiently promote translation of digital therapeutics into practice [72]. These designs can also help to answer important scientific questions related to mechanisms of impact and heterogeneity of effects (ie, what works for whom and how, what determinants are most associated with successful implementation in different settings). Systematic evaluation of relevant implementation outcomes and determinants across the stages of digital therapeutic research can iteratively inform directions for refinement of the digital intervention and targets for implementation support. Use of common measurement tools to evaluate implementation constructs across stages can facilitate study replication and cross-study comparisons and build cumulative knowledge to accelerate development of digital therapeutics that are maximally potent and implementable in systems of care,.

### Promote Academic-Industry Partnerships to Accelerate Evaluation and Translation of Digital Therapeutic Approaches for Behavioral Health

The workshop highlighted many examples of innovative and effective digital interventions but with limited broad adoption. There is a need for multidisciplinary teams that include a range of stakeholders (eg, patients, clinicians, researchers, technology developers, business experts, economists, and policy makers) to collaborate to develop effective and scalable digital therapeutics. The Food and Drug Administration, for example, has a new process for authorizing digital therapeutics, including for behavioral health (eg, reSET, Pear Therapeutics), that can serve as one model for fostering partnerships to propel translation.

There is also an abundance of behavior change apps in the commercial market space (ie, app stores) that may be helpful but have not been empirically evaluated. Unlike digital therapeutics developed within an academic research paradigm, commercial apps are often developed by business enterprises and commercial developers, with particular attention to promoting end-user engagement and broad dissemination. Initiatives to incentivize research teams to work with app developers to evaluate efficacy of commercially available apps can harness existing dissemination channels to promote scalability of evidence-supported digital therapeutics, particularly in direct-to-consumer markets (eg, app stores). An important component of such partnerships, however, is the ability to access internal usage data to understand how participants are using the apps. Since academic and industry stakeholders may have different motivations for a partnership, establishing clear guidelines for academic-industry partnerships at the outset to set expectations about issues such as roles, intellectual property, and access to and ownership of research-related usage data and algorithms may help to avoid later tensions and perceptions of conflict of interest for researchers.

### Identify Strategies to Promote Awareness About and Use of Science-Supported Digital Therapeutics

Participants acknowledged the potential usefulness of a curated space for useful apps for clinicians to recommend to their patients or clients. There are now evidence-supported rating schemas for mobile apps [73]. CTBH also supports a continuously updated curated repository of summaries of empirical studies of digital therapeutics for behavioral health [74], and other organizations are developing rating repositories for mobile apps for mental health conditions [75]. Studies are needed to determine how to facilitate broad adoption and sustainable implementation of existing evidence-supported digital therapeutics with patient populations in diverse care settings.

### Harness Big Data and Predictive Modeling Strategies to Promote Implementation Science for Digital Therapeutics

Recent studies highlight the potential of big data sources, such as Google and social media platforms (Twitter, Instagram), and innovative data analytic methods, such as deep neural networking, to predict substance use risk [19,20]. There is tremendous opportunity for collaborative research in the space of big data and novel analytics to identify individual needs and deliver personalized, “just-in-time” interventions using digital therapeutics. Social media platforms can be used to rapidly and inexpensively recruit representative patient and care setting stakeholders to test digital interventions as they are iteratively developed and to study optimal models for implementing digital therapeutics on these platforms.

### Address System Context Barriers

Common system-level barriers emerged in the workshop presentations. The interface of digital therapeutics with the array of electronic health records remains a significant barrier. To facilitate uptake of digital therapeutics within health systems, there is important work to be done to identify what information from digital therapeutics is clinically relevant to include in electronic health record systems, what information is best suited for access by end users as desired outside of the electronic health record, and how to optimize the intersection of digital therapeutics with electronic health records. A focus on alignment of digital therapeutics with the documentation needs of electronic health records early in development can accelerate translation.

Technology infrastructure also continues to be a source of significant access disparity, particularly in rural regions. Broadband is still not available in many rural regions of the United States, and mobile WiFi infrastructure is often unreliable. Federal and state initiatives to improve technology infrastructure in rural communities would help to reduce access disparities in these regions and promote opportunities for scaling digital behavioral health approaches to individuals in these often medically underserved areas.

### Conclusions

Digital therapeutic approaches to treatment of behavioral health conditions offer great potential for overcoming traditional barriers to widespread delivery of evidence-based care. Digital approaches have the potential to reach broad audiences with highly individualized care that can respond to an individual’s needs, preferences, culture, learning style, stage of recovery, and clinical trajectory over time. Individuals can control the pace of their treatment, and clinicians can augment their treatment practices with digital therapeutic tools to allow them to work at their highest level of training, to work with more patients or clients, and to focus more intensively on high-need clientele. This workshop highlighted important directions for digital behavioral health intervention research to promote broad adoption and optimize implementation of digital therapeutics for behavioral health conditions across diverse care settings and populations. CTBH, as an interdisciplinary community of researchers and practitioners, serves as an important resource to promote implementation science related to digital therapeutics. We encourage researchers and community and policy stakeholders to engage with us in this exciting work.

## Abbreviations

AI: American Indian
AN: Alaska Native
AUD: alcohol use disorder
CFIR: Consolidated Framework for Implementation Research
CTBH: The Center for Technology and Behavioral Health
D&I: Dissemination & Implementation
DMAIC: Define, Measure, Adapt, Improve, Control
LMIC: low- and middle-income countries
OIR: Outcomes of Implementation Research
SBIRT: screening, brief intervention, and referral to treatment
TES: Therapeutic Education System
